# Supplementary material for: Identification of Marine Biotechnology Value Chains with High Potential in the Northern Mediterranean Region
Source: Mar Drugs. 2023 Jul 22;21(7):416. doi: 10.3390/md21070416 (PMC10381324; doi:10.3390/md21070416)
Supplement: Supplementary file 1 [file marinedrugs-21-00416-s001.zip › Supplementary File S1.pdf]

Estimation of waste from the fish retail, commercial and processing industry

The following calculations have been made for the determination of fish by-products category 3 (FBP) from the processing of fishery and aquaculture products in the retail, commercial and processing chain, which are summarized in Table 2 of the paper. Many of the fishery and aquaculture products sold in supermarkets, public markets, neighbourhood fishmongers and central markets where seafood is sold are given to customers after being processed, i.e. the common "cleaning". "Cleaning", depending on the type of fish, may include gutting, removal of scales, skinning, filleting, gill removal, head removal, etc. After this treatment, about 15-30% is discarded (offal, scales, backbone and heads).

The biomass from this procedure is difficult to be estimated accurately. In the B-Blue Project Countries, the apparent consumption of fishery and aquaculture products (fish, molluscs and crustaceans in fresh, frozen or processed form) presented in Table 2 takes into account all forms consumed, even canned or smoked products, etc.

From the production data of Supplementary Table 1 (fisheries), Table 2 (fish produced from aquaculture), Table 3 (fish imports) and Table 4 (exports) the apparent consumption (Table 5) is calculated from the following formula:

Apparent consumption= Fish from fisheries production + Fish from aquaculture production + Imports of fish – Exports of fish.

The apparent per capita consumption is calculated if the apparent consumption will be divided with the population in each Country (Eurostat data) (Table 6).

Supplementary Table 1: Fisheries production-main species grouping in the B-Blue countries (in tons - live weight). Table A shows the Mediterranean production for France and Spain whereas Table B shows the total production of France and Spain. Portugal, although not a Mediterranean Country, is added in the total figures for the purpose of the B-Blue project.

A.

Country	Main species grouping	2014	2015	2016	2017	2018
**Croatia**	Fish	77,127	70,681	70,352	67,348	68,038
	Molluscs	2,426	2,332	1,968	1,441	1,151
	Crustaceans	765	873	933	1,084	1,180
**France** ***(Med Fisheries only)***	Fish	12,459	10,549	11,538	12,218	13,727
	Molluscs	2,409	2,008	2,336	2,401	2,858
	Crustaceans	131	140	355	357	290
**Greece**	Fish	50,408	54,199	61,332	63,267	62,726
	Molluscs	5,801	5,443	8,233	7,733	7,523
	Crustaceans	3,960	5,781	7,002	7,180	6,985
**Italy**	Fish	125,548	138,258	136,683	133,639	139,821
	Molluscs	38,389	38,264	39,362	39,807	42,283
	Crustaceans	18,699	21,037	20,690	22,648	23,549
**Montenegro**	Fish	1,290	1,219	1,324	1,011	1,199
	Molluscs	235	239	242	33	43
	Crustaceans	31	28	28	35	50

	Fish	162,275	168,456	165,856	160,943	158,334
**Portugal**	Molluscs	18,843	18,959	17,165	17,305	15,714
Mainly Atlantic fishery	Crustaceans	1,749	1,082	1,134	1,439	2,235
	Fish	370	315	271	256	229
**Slovenia**	Molluscs	38	26	33	23	34
	Crustaceans	2	2	7	1	1
	Fish	70,559	66,400	67,583	75,540	77,590
**Spain**	Molluscs	6,042	6,125	6,756	6,186	7,481
(Med Fisheries only)	Crustaceans	2,193	4,779	4,109	4,578	6,249
	**Fish**	500,036	510,077	514,939	514,222	521,664
**TOTAL**	**Molluscs**	74,183	73,396	76,095	74,929	77,087
	**Crustaceans**	27,530	33,722	34,258	37,322	40,539

B.

**Country**	**Main species grouping**	**2014**	**2015**	**2016**	**2017**	**2018**
	Fish	418,082	407,400	433,777	419,711	451,422
France	Molluscs	63,710	65,743	67,254	70,118	106,926
	Crustaceans	16,271	14,854	16,373	16,533	15,394
	Fish	968,713	899,740	859,394	887,747	871,863
Spain	Molluscs	74,979	61,422	39,880	46,631	37,070
	Crustaceans	18,423	13,696	13,366	14,701	16,088

Supplementary Table 2. Aquaculture (fish and shellfish) production in the B-BLUE countries (values in tons live weight). Figures refer to the whole Country production in France, Portugal and Spain and not only to the Mediterranean Sea.

**Country**	**Main species grouping**	**2014**	**2015**	**2016**	**2017**	**2018**
	Fish	12,403	14,773	15,042	15,052	17,132
**Croatia**	Molluscs	746	798	763	982	935
	Crustaceans	0	0	0	0	0
	Total	13,149	15,571	15,805	16,034	18,067
	Fish	44,654	38,700	48,690	47,816	42,953
**France**	Molluscs	135,573	124,487	131,767	140,628	144,096
	Crustaceans	58	62	50	57	44
	Total	180,285	163,249	180,507	188,501	187,093
	Fish	87,836	88,334	100,291	106,165	110,162
**Greece**	Molluscs	16,701	18,629	23,291	19,246	22,010
	Crustaceans	0	50	29	11	62
	Total	104,537	107,013	123,611	125,422	132,234
	Fish	48,341	48,402	56,633	56,634	50,163
**Italy**	Molluscs	100,374	100,345	100,345	100,345	93,171
	Crustaceans	15	16	22	21	5

	Total	148,730	148,763	157,000	157,000	143,339
**Montenegro**	Fish	680	624	737	808	852
	Molluscs	179	189	192	214	245
	Crustaceans	0	0	0	0	0
	Total	859	813	929	1,022	1,097
**Portugal**	Fish	6,488	4,782	4,851	5,362	4,556
	Molluscs	4,842	4,766	4,916	7,093	9,382
	Crustaceans	5	13	18	21	18
	Total	11,335	9,561	9,785	12,476	13,956
**Slovenia**	Fish	967	1,029	1,232	1,084	1,334
	Molluscs	430	588	627	666	609
	Crustaceans	0	0	0	0	0
	Total	1,397	1,617	1,859	1,750	1,943
**Spain**	Fish	59,533	61,810	64,111	66,591	60,535
	Molluscs	222,543	227,805	219,539	244,233	287,020
	Crustaceans	162	204	177	199	258
	Total	282,238	289,819	283,827	311,023	347,813

Supplementary Table 3. Imports of seafood (in tons).

**Country**	**2014**	**2015**	**2016**	**2017**	**2018**
Croatia	43,713	46,713	47,914	48,984	53,334
France	1,079,757	1,086,087	1,103,985	1,140,298	1,134,602
Greece	208,414	199,733	211,573	213,521	226,865
Italy	1,035,299	1,069,054	1,092,593	1,103,338	1,105,440
Montenegro	3,162	3,333	3,759	4,213	4,229
Portugal	475,478	477,588	502,846	523,319	512,323
Slovenia	18,455	19,788	22,818	22,499	22,501
Spain	1,578,864	1,625,846	1,673,254	1,729,472	1,708,859

Source: FAO

Supplementary Table 4. Exports of seafood (in tons).

**Country**	**2014**	**2015**	**2016**	**2017**	**2018**
Croatia	47,598	53,270	54,019	52,379	53,902
France	300,491	330,384	335,661	335,698	343,278
Greece	119,463	118,054	132,064	144,146	145,231
Italy	160,617	159,606	153,479	150,461	154,319
Montenegro	9	1	21	14	5
Portugal	268,700	272,916	247,669	259,971	261,906
Slovenia	6,000	6,890	11,352	9,052	8,683
Spain	1,101,402	1,105,226	1,104,597	1,173,009	1,191,664

Source: FAO

Supplementary Table 5: Estimation of apparent consumption (in Kg per person) for the determination of fish by-products (FBP) category 3 from the processing of fishery and aquaculture products, in the retail and processing chain. The population figures per year are as per ST6.

Country	2014	2015	2016	2017	2018
Croatia	18.34	16.77	17.14	16.98	18.23
France	15.67	15.04	15.61	15.90	16.19
Greece	19.47	19.33	20.82	20.48	22.14
Italy	11.82	12.17	12.83	13.10	13.30
Montenegro	7.02	7.08	7.81	8.04	8.27
Portugal	32.24	32.47	35.38	35.06	33.95
Slovenia	5.11	5.25	4.46	5.48	5.49
Spain	25.39	25.11	25.31	25.61	24.64

Supplementary Table 6. Population figures for the B-Blue Countries.

Country	2014	2015	2016	2017	2018
Croatia	4,246,809	4,225,316	4,190,669	4,154,213	4,105,493
France	66,165,980	66,458,153	66,638,391	66,809,816	67,026,224
Greece	10,926,807	10,858,018	10,783,748	10,768,193	10,741,165
Italy	60,782,668	60,795,612	60,665,551	60,589,445	60,483,973
Montenegro	621,521	622,099	622,218	622,387	622,359
Portugal	10,427,301	10,374,822	10,341,330	10,309,573	10,291,027
Slovenia	2,061,085	2,062,874	2,064,188	2,065,895	2,066,880
Spain	46,512,199	46,449,565	46,440,099	46,528,024	46,658,447

Source: EUROSTAT
